# High-density atrial mapping, P-wave analysis, and computational simulations in Brugada syndrome: Enhancing the understanding of atrial fibrillation

**DOI:** 10.1016/j.hroo.2025.06.027

**Published:** 2025-07-08

**Authors:** Beatrice Zanchi, Ali Gharaviri, Marco Bergonti, Simone Pezzuto, Tardu Özkartal, Maria Luce Caputo, Esther Scheirlynck, Carlo de Asmundis, Francesca Faraci, Giulio Conte

**Affiliations:** 1 Department of Innovative Technologies, Institute of Digital Technologies for Personalized Healthcare of SUPSI, Lugano, Switzerland; 2 Department of Quantitative Biomedicine, University of Zurich, Zurich, Switzerland; 3 Center for Computational Medicine in Cardiology, USI, Lugano, Switzerland; 4 Centre of Cardiovascular Science, University of Edinburgh, Edinburgh, United Kingdom; 5 Heart Rhythm Management Centre, Postgraduate Program in Cardiac Electrophysiology and Pacing, European Reference Networks Guard-Heart, Universitair Ziekenhuis Brussel–Vrije Universiteit Brussel, Brussels, Belgium; 6 Department of Electronics and Informatics (ETRO), Vrije Universiteit Brussel (VUB), Brussels, Belgium; 7 Division of Cardiology, Cardiocentro Ticino Institute, Ente Ospedaliero Cantonale, Lugano, Switzerland; 8 Laboratory of Mathematics for Medicine and Biology, Department of Mathematics, University of Trento, Trento, Italy; 9 Faculty of Biomedical Sciences, Università della Svizzera Italiana (USI), Lugano, Switzerland

**Keywords:** Brugada syndrome, Computer simulations, Cardiac modeling, Electroanatomic mapping, Atrial fibrillation, P wave, Electrocardiogram

## Abstract

**Background:**

An increased risk of atrial fibrillation (AF) has been reported in patients with Brugada syndrome (BrS). The pathophysiology of AF susceptibility in BrS is largely unknown.

**Objective:**

This study aimed to characterize the atrial electrical properties of patients with BrS with and without AF based on P-wave and high-density atrial mapping analysis with a focus on conduction velocity (CV) and provide mechanistic insights on AF susceptibility using computer modeling.

**Methods:**

Electrocardiographic signals were processed, and P-wave parameters were analyzed in a consecutive series of patients with and without BrS. High-density atrial mapping was performed in patients with BrS undergoing an electrophysiological procedure. CV vectors were numerically approximated at each recording point using polynomial surface fitting. AF initiation susceptibility was simulated in a 3-dimensional atrial model and compared with control simulations.

**Results:**

A total of 133 subjects (89 patients with BrS and 44 controls) were included. AF history was present in 11% of patients with BrS. Patients with BrS had longer mean P-wave duration than controls (135 ms vs 124 ms, *P* < .01), whereas no P-wave parameter was able to discriminate between patients with BrS with and without AF. CVs correlated with total atrial activation time (TAAT) (R^2^ = 0.706), and TAATs mildly correlated with P-wave duration (R^2^ = 0.12). A significantly higher conduction pattern complexity, quantified as the number of coexisting fibrillation waves, was observed in BrS than in control simulations. In all simulations, regardless of the degree of fibrosis, AF initiation rates were significantly higher in BrS than in control simulations.

**Conclusion:**

Conventional P-wave parameters do not identify patients with BrS prone to AF. Increased TAAT is related to reduced local CVs, explaining the prolonged P-wave duration observed in patients with BrS. Simulation studies showed significantly higher AF susceptibility initiation in patients with BrS than in controls.

## Graphical abstract

**Figure:**
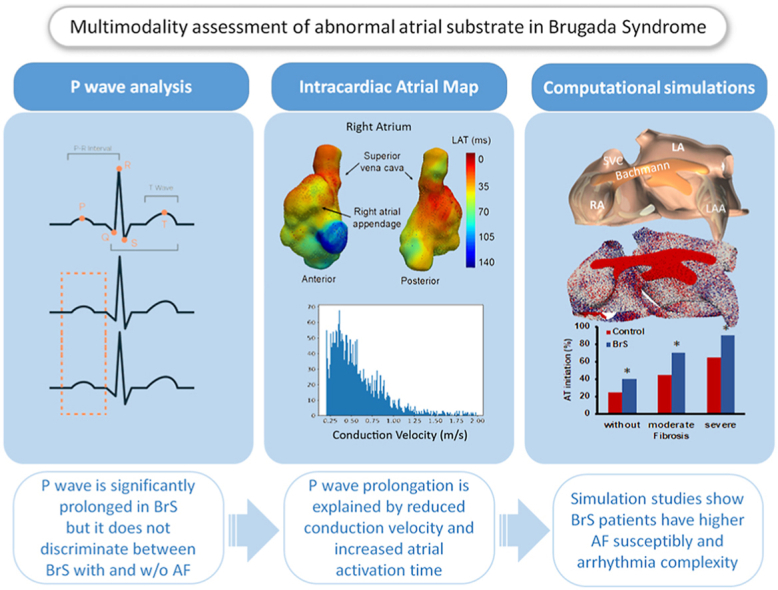

**Key Findings**

▪ Patients with Brugada syndrome (BrS) present marked P-wave parameter abnormalities, including prolonged P-wave duration.
▪ P-wave parameters cannot identify patients with BrS prone to atrial fibrillation (AF).
▪ Total atrial activation time, documented at high-density atrial mapping, correlates positively with P-wave prolongation; however, the correlation is weak possibly suggesting the presence of other factors contributing to prolonged P-wave duration.
▪ Simulation studies show that BrS atrial models have a higher AF susceptibility and arrhythmia complexity, regardless of the presence and degree of atrial fibrosis.

## Introduction

Brugada syndrome (BrS) is a rare cardiovascular disease, associated with a genetically determined ion channel dysfunction and an increased risk of ventricular arrhythmias (VAs).^1^, ^2^, ^3^

Despite the electrocardiographic (ECG) ventricular hallmark, patients with BrS can present with a concealed abnormal atrial ECG phenotype, characterized by P-wave abnormalities, and a substantial risk of atrial fibrillation (AF).^4^, ^5^, ^6^, ^7^ Recently, an artificial intelligence–guided approach, based only on P-wave characteristics, has been proposed to enhance BrS diagnosis.^8^ Furthermore, a noninvasive ECG imaging study has reported significantly prolonged total atrial conduction time and local atrial conduction time in 43 patients with BrS compared with 40 control patients.^9^

A full understanding of the pathophysiological mechanisms leading to the abnormal atrial phenotype and consequently to AF susceptibility in these patients is still missing. Previous computational studies aimed at investigating the main factors contributing to the development of VAs, whereas no computational atrial models have investigated the abnormal atrial phenotype of BrS so far.^10^, ^11^, ^12^

This study aimed to (1) characterize P-wave parameter abnormalities in patients with BrS with and without AF, (2) assess atrial electrical properties (total atrial activation time [TAAT] and conduction velocities [CVs]) in patients undergoing high-density atrial mapping (HDAM), and (3) evaluate AF initiation susceptibility and complexity using computer modeling and simulations.

## Methods

### Study population

The study population included (1) a retrospective series of patients with BrS identified at Cardiocentro Ticino Institute, EOC, Lugano, Switzerland, and (2) a prospective cohort of patients with BrS undergoing HDAM. Moreover, a control group of healthy individuals referred for suspected BrS was included. All the subjects included in the control group were asymptomatic family members evaluated in the context of family screening for BrS and did not display any structural or electrophysiological (EP) cardiac abnormality. Moreover, they all underwent an ajmaline challenge that did not unmask a diagnostic Brugada type 1 ECG.

Institutional and ethics committee approval and patient consent were obtained (Swiss Ethics, approval number: BASEC 2019-00754). The research reported adheres to relevant ethical guidelines (ie, the Declaration of Helsinki). The diagnosis of BrS was established based on the current guidelines and the Shanghai Score System criteria.^2^ An ECG was considered diagnostic of BrS only if a coved-type ST elevation of ≥2 mm was documented in ≥1 lead from V1 to V3 in the presence or absence of a sodium (Na^+^) channel blocker agent. Medical and family history evaluation, physical examination, bidimensional transthoracic echocardiography, cardiac magnetic resonance imaging, and genetic testing were performed in patients with BrS.

### ECG acquisition and preprocessing

All patients with BrS and control group subjects underwent a 12-lead ECG recording obtained in sinus rhythm by a high-resolution ECG machine (CARDIOVIT CS-200 Excellence; Schiller) having a sampling frequency of 1 kHz and a band-pass filter with cutoff frequencies set at 0.5–300 Hz. Each recording had a duration of a minimum of 30 seconds to a maximum of 5 minutes per patient. Recordings were automatically filtered using a line frequency filter for the suppression of superimposed 50- or 60-Hz sinusoidal interference through adaptive digital filtering. To remove the remaining noise, the signals were filtered by using a fifth-order Chebyshev low-pass filter with a cutoff frequency set to 100 Hz. Similarly with previous studies, the extraction of P-wave indexes was done on averaged P waves.^7^^,^^8^ The filtered signal was divided into 15-second epochs, and all the valid P waves were then identified. At the end of the windowing process, multiple 15-second epochs per patient were obtained. Segmentation of the original signal into 15-second epochs was performed for 2 key reasons: (1) to enhance the quality of the averaged P-wave and, consequently, to improve the precision of P-wave features extraction by reducing noise and artifacts and (2) to capture the full spectrum of ECG variability, given that these signals can exhibit dynamic changes (eg, heart rate variability). To isolate P waves in each 15-second epoch, we first detected the R peak with a variation of the Pan-Tompkins algorithm.^13^ This algorithm acts as a high-pass filter, highlighting the high-frequency QRS complexes. P waves were then extracted using a 300-ms window starting 350 ms before each R peak. To construct a robust P-wave template, all valid P waves within the epoch were evaluated. Each of them was temporarily considered a candidate template and compared with the others using cross-correlation. The P wave with the highest average correlation across the set was selected as the final template for that epoch. P waves with poor correlation (eg, <0.9) to the selected template were discarded to exclude noisy or ectopic beats. The remaining P waves were temporally aligned to the template based on the lag of maximum correlation and then averaged to produce a clean, representative waveform for feature extraction. This process was performed independently for each ECG lead.

### P-wave features extraction

For each patient, we considered 60 local features (5 features per 12 leads) and 5 global features. Global features include the average P-wave duration, PR interval, P-wave terminal force in lead V1, full width at half maximum (FWHM), and P-wave axis. In particular, prolonged P-wave duration and P-wave terminal force in lead V1 indicate left atrial (LA) depolarization impairment. FWHM is the time interval where the P-wave exceeds half of its maximum amplitude, marking P-wave amplitude dispersion over time.

Local features are extracted individually from each of the 12 ECG leads, with the following 5 features: P-wave area, number of P-wave peaks, maximum P-wave amplitude, P-wave entropy, and P-wave sample entropy. Given that these characteristics are extracted from each ECG lead, the total number of local features is 60. P-wave amplitude indicates the maximum electrical force during the stimulus propagation between atrial chambers. P-wave entropy measures the signal’s amplitude profile uncertainty over time, indicating the repeatability of certain patterns within the averaged P-wave. Entropy and sample entropy are computed as previously reported.^5^^,^^7^ A summary of P-wave features is presented in Table 1.

**Table 1:** P-wave indices

Feature type	Feature name	Definition
Global	P-wave duration	Time between the onset and the offset of the P wave across leads
Global	PR interval	Time from the onset of the P wave to the start of the QRS complex
Global	PTFV1	The terminal negative area of the P wave in lead V1 reflects left atrial abnormality
Global	FWHM	Time during which the P wave exceeds 50% of its maximum amplitude
Global	P-wave axis	Direction of atrial depolarization in the frontal plane
Local (per ECG lead)	P-wave area	Area under the P-wave curve in each lead
Local (per ECG lead)	Maximum amplitude	Maximum absolute amplitude of the P wave
Local (per ECG lead)	Number of peaks	Count of distinct local maxima within the P wave
Local (per ECG lead)	P-wave entropy	Measures signal complexity using amplitude variations^5^^,^^7^
Local (per ECG lead)	P-wave sample entropy	Refined entropy metric estimating self-similarity and predictability in the signal

ECG = electrocardiogram; FWHM = full width at half maximum; PTFV1 = P-wave terminal force in lead V1.

### EP procedure with HDAM

Electroanatomic HDAM was performed in a prospective cohort of patients with BrS undergoing, between 2019 and 2020, an EP procedure (programmed ventricular stimulation for risk stratification of sudden cardiac death or AF ablation).

HDAM was performed using an electroanatomic mapping system and a 64-electrode basket mapping catheter (Rhythmia HDx mapping system and IntellaMap ORION mapping catheter, Boston Scientific, Cambridge, MA) or a 20-electrode catheter (CARTO 3 mapping system and Pentaray mapping catheter, Biosense Webster, Diamond Bar, CA). Right atrial (RA) mapping was systematically performed in patients with BrS undergoing an EP study or an ablation procedure. LA mapping was performed only in patients with BrS undergoing AF ablation. LA access was not performed in patients with BrS without a clinical indication nor in control subjects. All patients were in sinus rhythm during HDAM.

### Local activation detection

Unipolar electrograms, electrode locations, and RA/LA geometry were imported into MATLAB (MathWorks, Natick, MA) and analyzed offline using OpenEP (version 1.0.3), an open-source software.^14^ Electrograms were recorded with a sampling frequency of 953.6 Hz. All recorded signals were visually inspected, and signals and electrograms of low quality were removed from the data. Thereafter, the signals that remained in the dataset were detrended and denoised using a second-order Savitzky-Golay filter with a span of 201 samples and a fifth-order band-pass Butterworth filter, respectively. The local activation time (LAT) in each unipolar electrogram was calculated as the time interval between the defined reference time, the activation time recorded at the coronary sinus electrode, and the time of maximum negative slope in the voltage. LATs detected at each electrode were projected onto the closest vertex on the surface mesh, with multiple LATs at a shared vertex averaged to obtain a single value. A 2-mm neighborhood check was used to detect and automatically exclude LAT outliers. LAT maps on the surface mesh were reconstructed using the radial basis function interpolation method. A Gaussian kernel was used for the radial basis function interpolation, and the hyperparameters, including epsilon and the smoothing parameter, were carefully optimized to ensure the most accurate interpolation.

### TAAT

Total atrial activation was calculated by defining the time difference between the identified earliest LAT within the RA and the activation time detected upon conduction to the distal electrode in the coronary sinus catheter. This method provides an approximate measure of the TAAT.

## CV calculation

CV vectors were numerically approximated at each recording point using a well-established and previously published method, polynomial surface fitting.^15^

In brief, we fitted a polynomial surface, using a standard least squares algorithm, through a subset of electrode coordinates and LATs.

The inverse gradient of the fitted polynomial surface was used to approximate the CV vector at each recording point. To increase the accuracy of calculated CVs, artificial high CVs caused by wave collision sites were identified and excluded from the data. These collision sites were located by obtaining the divergence of the CV vector field with values of less than –1.5 s^-1^.^16^ After excluding CVs in the area of wave collisions, calculated localized CVs were interpolated on the surface mesh using the radial basis functions.

### Computer simulations

The simulations were performed on a highly detailed 3-dimensional (3D) model of the human atria extensively described previously.^17^, ^18^, ^19^ Action potential propagation was simulated with the monodomain reaction-diffusion equation using the Courtemanche-Ramirez-Nattel model for the ionic currents. AF-induced changes in ionic currents were incorporated by setting the conductivities for transient outward potassium current, L-type calcium current, and inward rectifier potassium current at 40%, 35%, and 200% of their normal values, respectively.^19^ To explore the arrhythmogenic impact of localized and heterogeneous loss of function of Na^+^ ion channel, caused by *SCN5A* variants, we simulated a 70% reduction in Na^+^ conductivity and applied it only to 50% of randomly selected model elements. The impact of structural remodeling was replicated by introducing 3 scenarios of fibrosis: no fibrosis, moderate fibrosis, and severe fibrosis, respectively emulated by designating 0%, 50%, and 70% of randomly selected elements in the model as fibrotic.^19^ Within the model, fibrotic elements were electrically decoupled transversally to the fiber direction.

AF was initiated in each simulation by a train of stimuli that lasted 2.5 seconds with a progressive reduction in pacing intervals applied to 20 different pacing locations. AF initiation was considered successful if the fibrillation activity remained sustained during the whole period of simulation. The effect of BrS channelopathy on AF initiation susceptibility was compared with control simulations with no BrS Na^+^ channel remodeling and in the presence of different degrees of fibrosis. The average number of waves over time was used as a surrogate parameter to quantify the complexity of conduction patterns during fibrillation. A wave was defined as a contiguous region in which all nodes exhibited transmembrane voltages exceeding a threshold of −60 mV. The number of waves was computed at every millisecond of simulated time and averaged over the period after pacing, up to the end of the simulation.

### Statistics

Continuous data are presented as mean ± standard deviation and compared using the 2-sided Student *t* test. Categorical data are delineated as numerical counts and their respective percentages relative to the total population. In the simulation studies, we used a Student *t* test to examine the statistical differences between the groups. In addition, to ensure the robustness and validity of the results, the Bonferroni correction test was conducted to further analyze pairwise differences among multiple groups when significant differences were detected. Their comparative analysis is conducted using the χ^2^ and Fisher exact tests. Pearson correlation was used to establish the degree of correlation between the variables. Significance is attributed to *P* < .05, denoting statistical significance. Statistical analyses were performed with SciPy-based library (version 1.10.1; Python Software Foundation, Wilmington, DE).

## Results

### Study population

A total of 133 subjects (89 patients with BrS and 44 healthy controls) were included. Clinical features are presented in Table 2. Of 89 patients with BrS (68% men, mean age 47 ± 14 years, *SCN5A* pathogenic/likely pathogenic [P/LP] variant 12.5%), 10 (11.2%) presented with previous episodes of AF. Six of them (60%) were men, and 2 (20%) presented with a spontaneous type 1 ECG.

**Table 2:** Study population characteristics

Clinical features	Patients with Brugada syndrome (n = 89)	Negative ajmaline subjects (n = 44)	*P* value
Male sex, n (%)	61 (68.5%)	28 (63.6%)	.87
Age (y)	47 ± 14	46 ± 14	.95
Family history of SCD, n (%)	18 (20.2%)	4 (9.1%)	.15
Syncope, n (%)	19 (21.3%)	8 (18.2%)	.68
Previous sustained VAs, n (%)	7 (7.8%)	-	-
Previous ICD implantation, n (%)	19 (21.3%)	-	-
* SCN5A* P/LP variant, n (%)	11 (12.3%)	-	-
Sustained AF, n (%)	10 (12.6%)	-	
Heart rate (bpm)	81 ± 13	78 ± 10	.73
Echocardiographic parameters			
LVEF (%)	60.7 *±* 3.4	60.7 ± 2.8	.80
LA diameter (mm)	30.7 ± 5.7	30.7 ± 6	.49
LAVI (mL/m^2^)	24.8 ± 5.3	24.8 ± 7.8	.82
RAVI (mL/m^2^)	21.3 ± 4.6	21.35 ± 6.6	.22
Brugada ECG pattern, n (%)			
Spontaneous Brugada type 1	25 (28%)	-	**-**
Ajmaline-induced Brugada type 1	55 (70.8%)	-	**-**
Fever-induced Brugada type 1	1 (1.1%)	-	**-**
P-wave parameters (ms)			
PR interval	177.4 ± 24.8	170.3 ± 21.9	.09
P-wave duration	135.5 ± 17.3	124.2 ± 15.7	<.01
QRS interval	98.2 ± 5.3	96.2 ± 3.3	.76

AF = atrial fibrillation; bpm = beats per minute; ECG = electrocardiogram; ICD = implantable cardioverter-defibrillator; LA = left atrium; LAVI = left atrial volume index; LVEF = left ventricular ejection fraction; P/LP = pathogenic/likely pathogenic; RAVI = right atrial volume index; SCD = sudden cardiac death; VA = ventricular arrhythmia.

### P-wave analysis in patients with BrS vs healthy controls

A detailed comparison of P-wave features between patients with BrS and controls and between patients with BrS with and without AF is presented in Supplemental Tables 1 and 2, respectively. Patients with BrS presented more pronounced P-wave abnormalities (longer P-wave duration, P-wave area, FWHM, and terminal force in V1) than control subjects. None of the P-wave parameters was able to identify patients with BrS with a history of AF.

### HDAM

**Figure 1:**
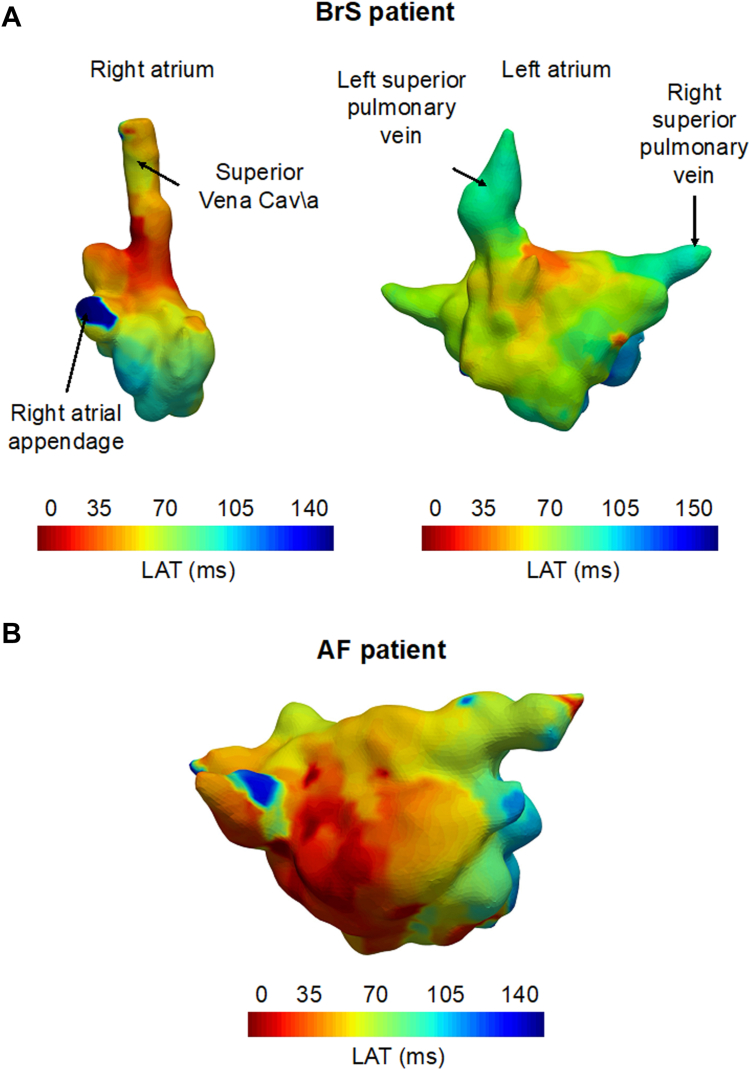
LAT map in a patient with BrS. **A:** An example of a constructed LAT map of a patient with BrS (left, right atrium; right, left atrium). **B**: An example of a constructed LAT map of the left atrium of a patient with AF. AF = atrial fibrillation; BrS = Brugada syndrome; LAT = local activation time.

**Figure 2:**
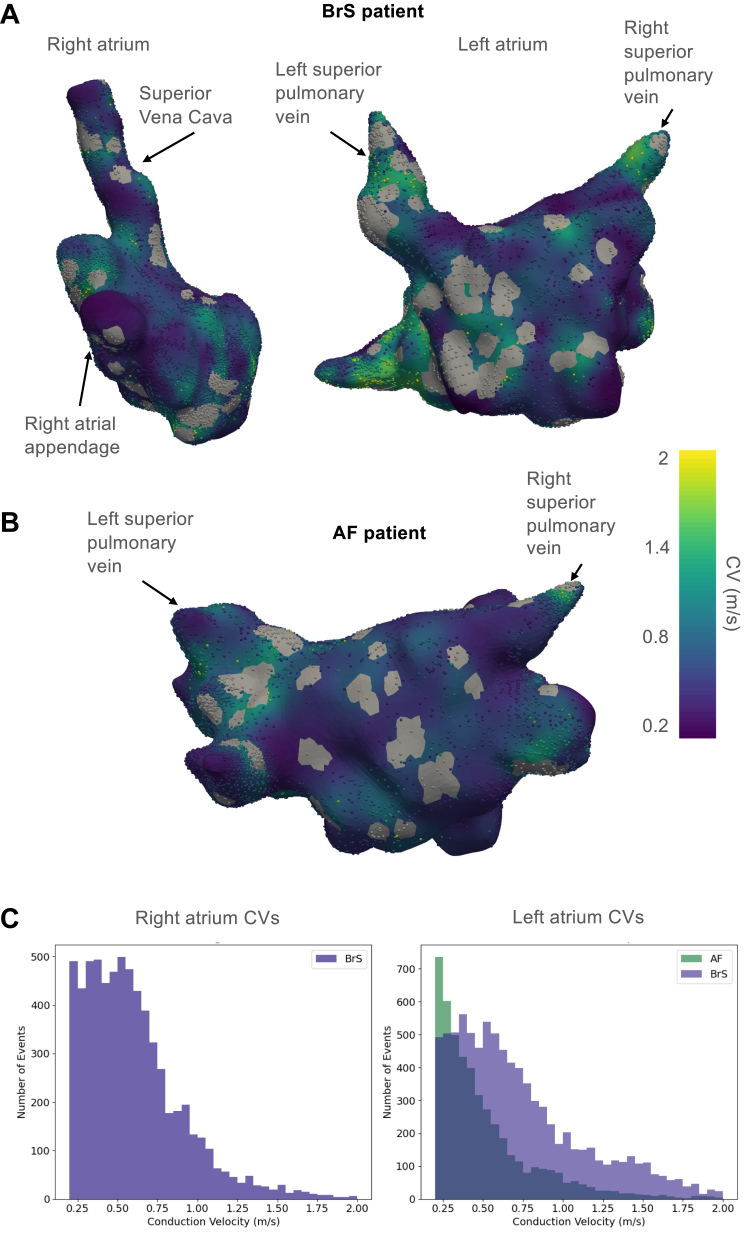
Corresponding CV maps and histograms. **A:** Corresponding CV of a patient with BrS (left, right atrium; right, left atrium). **B:** Calculated CV of the left atrium of the patient with AF. **C**: Corresponding CV histograms. AF = atrial fibrillation; BrS = Brugada syndrome; CV = conduction velocity.

A total of 15 patients with BrS (mean age 43 years, males 87%, spontaneous type 1 ECG 27%, *SCN5A* P/LP variant 13%) underwent HDAM. Clinical characteristics of patients with HDAM are presented in Table 3. A total of 74,785 unipolar voltage points were obtained during HDAM with an average of 5734 ± 201 points per patient. Of them, a mean of 1402 ± 181 points were <0.5 mV (1.9%). The mean bipolar voltage was 1.94 ± 0.48 mV. Examples of the calculated LATs interpolated on the RA and LA surface meshes of a patient with BrS and the LA surface of a patient with AF without BrS are shown in Figure 1A and 1B, respectively. The corresponding localized CVs interpolated on the surfaces for both patients with BrS and AF are shown in Figure 2A and 2B. Gray areas in the CV maps indicate regions where data were excluded owing to insufficient data points for reliable CV calculation or the occurrence of wave collisions. CV histograms of the localized CVs for the RA and LA of the patient with BrS and the LA of the patient with AF are presented in Figure 2C. The mean and median CVs for patients with BrS undergoing HDAM are presented in Table 3. Across all patients, most calculated CVs were skewed toward the left (slow) side of the CV histogram, with median CVs ranging from 0.31 m/s to 0.49 m/s and interquartile ranges between 0.39 m/s and 0.64 m/s.

**Table 3:** Clinical features and calculated CVs in patients undergoing HDAM

Patient	Age	Sex	Brugada type 1 ECG	Genetic test result	Spontaneous AF	PVI	LA diameter	P-wave duration (ms)	CV (m/s), median	CV (m/s), mean
#1	38	M	Ajmaline induced	Negative	Yes	Yes	38 mm (LAVI 24 mL/m^2^)	163	0.4942	0.5714
#2	54	M	Ajmaline induced	SCN5A LP variant	No	No	36 mm (LAVI: 22 mL/m^2^)	146	0.5746	0.49
#3	53	M	Spontaneous	Negative	No	No	37 mm (LAVI: 25 mL/m^2^)	159	0.3629	0.4464
#4	48	M	Ajmaline induced	Negative	No	No	30 mm (LAVI: 27 mL/m^2^)	117	0.47	0.55
#5	50	M	Ajmaline induced	Negative	No	No	38 mm (LAVI: 29 mL/m^2^)	136	0.5125	0.6056
#6	27	F	Ajmaline induced	Negative	No	No	33 mm (LAVI: 22 mL/m^2^)	93	0.5326	0.6239
#7	27	F	Ajmaline induced	Negative	No	No	23 mm (LAVI: 21 mL/m^2^)	131	0.2907	0.3738
#8	46	M	Spontaneous	Negative	No	No	31 mm (LAVI: 21 mL/m^2^)	122	0.4476	0.5243
#9	45	M	Ajmaline induced	Negative	No	No	36 mm (LAVI: 30 mL/m^2^)	148	0.56	0.6414
#10	57	M	Ajmaline induced	Negative	No	No	32 mm (LAVI: 30 mL/m^2^)	155	0.3637	0.45
#11	53	M	Ajmaline induced	Negative	Yes	Yes	49 mm (LAVI: 55 mL/m^2^)	95	0.54	0.61
#12	45	M	Spontaneous	Negative	No	No	36 mm (LAVI: 25 mL/m^2^)	102	0.56	0.64
#13	31	M	Spontaneous	Negative	No	No	35 mm (LAVI: 30 mL/m^2^)	110	0.47	0.55
#14	46	M	Fever induced	*SCN5A* LP variant	No	No	40 mm (LAVI: 22 mL/m^2^)	97	0.44	0.53
#15	34	M	Ajmaline induced	Negative	Yes	Yes	35 mm (LAVI: 30 mL/m^2^)	101	0.31	0.338

AF = atrial fibrillation; CV = conduction velocity; ECG = electrocardiogram; F = female; HDAM = high-density atrial mapping; LA = left atrium; LAVI = left atrial volume index; LP = likely pathogenic; M = male; PVI = pulmonary vein isolation.

### TAAT, P-wave duration, and local CV

**Figure 3:**
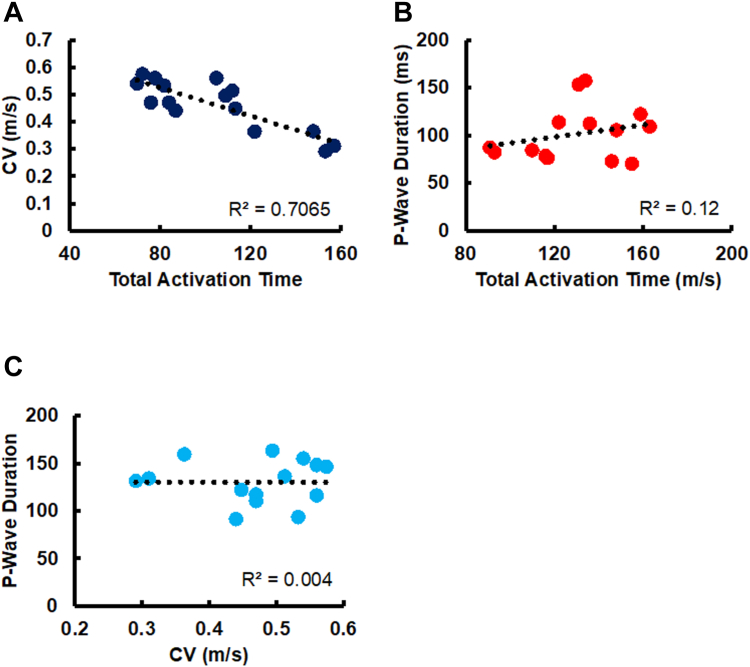
Relationship among calculated CVs, total atrial activation time, and P-wave duration in patients with Brugada syndrome undergoing high-density atrial mapping. **A:** CV median vs atrial total activation time**. B:** Averaged P-wave duration versus atrial total activation time. CV = conduction velocity.

In Figure 3, the median of calculated local CVs is plotted against TAAT. A negative correlation (R^2^ = 0.706) between the CV medians and TAATs was observed, given that lower CVs led to longer time intervals in which the atria were completely activated. Moreover, a positive but weaker correlation (R^2^ = 0.12) between P-wave durations and TAATs was detected.

### Computer simulations

**Figure 4:**
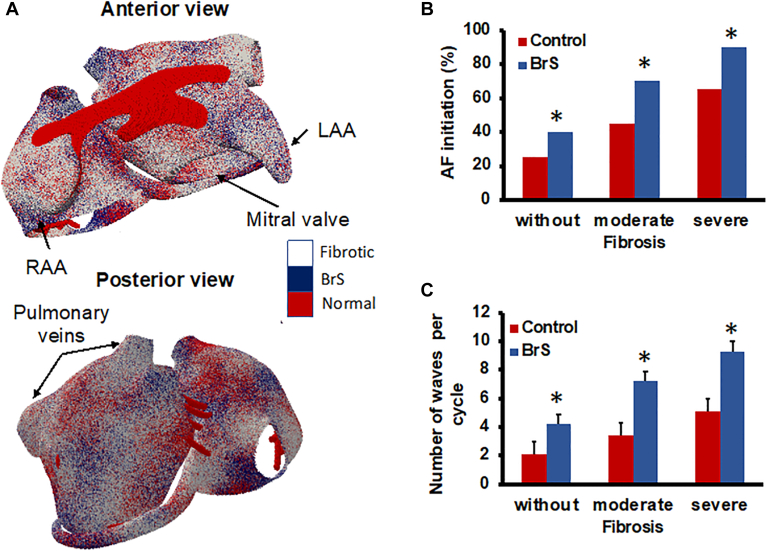
Simulation studies on AF susceptibility and arrhythmia complexity in patients with BrS. **A:** Visualization of random BrS channelopathies and fibrosis distribution in the atrial model, used for the simulations (top row, anterior view; bottom row, posterior view). **B:** AF initiation rate. **C:** Number of fibrillation waves. AF = atrial fibrillation; BrS = Brugada syndrome; LAA = left atrial appendage; RAA = right atrial appendage.

In all simulations, regardless of the fibrosis degree, AF initiation rates were significantly higher in BrS simulations than in control simulations. A significantly higher conduction pattern complexity, quantified as the number of coexisting fibrillation waves, was observed in BrS simulations compared with control simulations (Figure 4).

## Discussion

To the best of our knowledge, this is the first study that has systematically evaluated atrial electrical abnormalities in patients with BrS with and without a history of AF using advanced ECG postprocessing techniques, HDAM, and simulation studies by atrial computer modeling to mechanistically elucidate the observed abnormalities and simulate AF initiation susceptibility.

The main findings of our analysis are the following: (1) patients with BrS presented marked P-wave parameter abnormalities, including prolonged P-wave duration, which could not discriminate patients with and without history of AF; (2) HDAM shows a correlation between increased TAAT and decreased local CVs; (3) TAAT correlates positively with P-wave prolongation, but the correlation is weak possibly suggesting the presence of other factors contributing to prolonged P-wave duration; and (4) simulation studies show that BrS atrial models have a higher AF susceptibility and arrhythmia complexity, regardless of the presence and degree of atrial fibrosis.

### P-wave parameter abnormalities in patients with BrS with and without a history of AF

The baseline ECG of patients with BrS can reflect atrial electrical abnormalities resulting from the presence of an atrial channelopathy independently from the presence of previous episodes of AF.^5^^,^^7^^,^^8^ Indeed, it has been previously reported that patients with BrS and no history of AF present with abnormal P-wave parameters, including P-wave prolongation.^7^ Of note, the atrial ECG phenotype can be detected even in the absence of spontaneous type I ECG, symptoms, and *SCN5A* P/LP variants.^7^ Our study confirms previous findings from a larger population of patients with BrS without a history of AF. In these patients, an abnormal ECG atrial phenotype, characterized by increased P-wave duration, was consistently detected, suggesting the presence of an atrial EP abnormality despite the absence of AF.

P waves on the surface ECG represent atrial depolarization, and their duration may be a reliable noninvasive measurement of atrial conduction time. P-wave duration has already been reported as an effective tool to identify patients prone to paroxysmal AF, given that it enables discriminating patients with a history of AF and no overt structural cardiac abnormalities from healthy age-matched subjects.^20^ In contrast, in our study, none of the conventional P-wave parameters were found to be associated with an increased risk of AF in patients with BrS. This finding raises the question of the need for novel, reliable atrial ECG parameters able to identify patients with BrS at risk of AF.

Previous literature on interatrial block (IAB) and AF/stroke risk has primarily focused on P-wave duration and morphology in the inferior leads (II, III, aVF) as simple markers of atrial conduction abnormalities.^21^^,^^22^ Our study included conventional P-wave parameters from all leads, including the inferior leads, but also extended the analysis to a broader set of advanced ECG features not limited to traditional IAB. Moreover, our study cohort—adults with BrS—is different from the patient population typically investigated in IAB/AF/stroke risk studies. Therefore, the absence of an association between conventional P-wave parameters and AF in our cohort should be interpreted with caution. Whether the IAB criteria hold predictive value in the specific EP substrate of patients with BrS should be further explored.

Our results on the correlation between P-wave duration and TAAT are in line with a study by Bisignani et al,^9^ which investigated atrial abnormalities using ECG imaging in patients with BrS. The study reported a significantly prolonged TAAT in patients with BrS compared with the control population without BrS or structural heart disease.

### HDAM of the abnormal atrial phenotype of BrS

The arrhythmogenic substrate of BrS is not restricted to the ventricular level. Similar alterations at the atrial level could be the substrate for reentrant atrial tachyarrhythmias.^4,5^

The atrial anomaly might be caused by the prolonged atrial action potential and increased intra-atrial conduction time as reported by Kusano et al,^4^ who investigated differences in EP parameters between patients with BrS with and without AF. They reported, among various EP parameters, a significantly increased interatrial conduction delay in patients with BrS with AF, indicating the presence of an impaired global conduction of the atrial myocardium.

No specific studies have been conducted so far on HDAM in patients with BrS. Lambiase et al^23^ reported a high-density ventricular substrate mapping in BrS showing marked regional endocardial conduction delay and heterogeneity in repolarization.

Our study is the first assessment based on HDAM findings reporting on local CV and TAAT in BrS. Patients with BrS presented with longer TAATs, explained by lower local CVs. An increased duration of P waves was only partially explained by an increased TAAT. This suggests the presence of other intrinsic or modulating factors contributing to P-wave prolongation in BrS.

### Computational studies to investigate AF susceptibility in BrS

AF is not an uncommon finding in patients with BrS and can lead to inappropriate therapies in patients with an implantable cardioverter-defibrillator.^24^^,^^25^ The pathophysiological mechanism of AF in patients with BrS is still unknown, and although the role of pulmonary vein triggering is well established, AF maintenance in these patients with generally normal atrial substrate remains a matter of debate. Moreover, long-term results of AF ablation in patients with BrS are relatively poor compared with age-related patients with lone AF.^6^ AF development in these patients might be related to functional or structural cellular atrial abnormalities rather than to pulmonary vein ectopic triggering activity. This implies the need for a different strategy in AF ablation procedures and a different target for pharmacologic therapies.

Computational models of cardiac EP are well established and perfectly complement clinical and experimental data. Previous computational studies have aimed at investigating the main factors contributing to the development of VAs, and no specific atrial 3D computational models have been developed to assess AF susceptibility in BrS.^10^, ^11^, ^12^ Our study is the first to use an atrial 3D computational model to explore susceptibility to AF in this population. In a highly detailed human atrial model incorporating realistic EP properties and fiber architecture, we simulated the effects of BrS-related Na^+^ channel dysfunction and observed increased AF susceptibility and arrhythmia complexity—even in the absence of atrial fibrosis. To mimic the EP substrate associated with BrS, we imposed a 70% reduction in Na^+^ current conductance in 50% of model elements. This approach was not intended to replicate a specific genotype, but rather to represent the spatial heterogeneity of Na^+^ channel dysfunction reported in patients with BrS, as seen in both experimental mapping and clinical imaging studies. Our goal was to explore how localized loss of excitability may contribute to arrhythmogenesis in structurally intact tissue. Notably, a comparable degree of Na^+^ current reduction has been used in a previous whole-heart computational model of BrS to successfully reproduce key EP features, including altered conduction and ECG patterns, lending further support to the plausibility of our assumptions.^12^

Patient-specific or personalized computational approaches may be valuable in the future to improve the clinical outcome of AF ablation strategies in patients with inherited ion channel disorders. Ideally, computational models could be further individualized by incorporating patient-specific characteristics associated with a specific gene variant.

### Limitations

Our study has a certain number of limitations. It is a single-center retrospective study conducted, owing to the rarity of the condition, in a small population of adult patients with heterogeneous clinical characteristics. The number of patients with AF was limited; therefore, comparison analysis results between patients with BrS with and without AF need to be validated in larger cohorts. Moreover, HDAM was not performed in all cases. In addition, although the atrial 3D computational model used in our simulations is anatomically detailed, it is based on a generic atrial geometry and does not incorporate patient-specific anatomic variability, which may play a role in AF initiation and propagation dynamics. The fibrosis patterns implemented in the model were also not directly derived from clinical imaging data but were designed to explore the interaction between BrS-related EP remodeling and varying degrees of structural remodeling (mild to severe) under controlled conditions. The Na^+^ channel conductance reduction to simulate BrS-related EP changes may not be fully representative of biological reality, given that experimental and clinical evidence quantifying Na^+^ channel loss in human atrial tissue remains limited. Finally, the model did not assess the relative contributions of specific genetic variants or modulating physiological factors such as sex hormones and autonomic tone, which may influence AF susceptibility and complexity in patients with BrS.

## Conclusion

Although patients with BrS present P-wave parameter abnormalities, including prolonged P-wave duration, these parameters cannot identify patients with BrS prone to AF. Increased TAAT and reduced local CVs may explain the prolonged P-wave duration. Simulation studies show that BrS atrial models have a higher AF susceptibility and arrhythmia complexity than controls, regardless of the presence and degree of atrial fibrosis. However, the multifactorial nature of BrS (S*CN5A-*related atrial substrate alterations and modulating factors) may have an impact on the pathogenesis of AF, and this combination in computational studies remains to be determined.
