# Supplementary material for: High-density atrial mapping, P-wave analysis, and computational simulations in Brugada syndrome: Enhancing the understanding of atrial fibrillation
Source: Heart Rhythm O2. 2025 Jul 8;6(10):1621–31. doi: 10.1016/j.hroo.2025.06.027 (PMC12570171; doi:10.1016/j.hroo.2025.06.027)
Supplement: Supplemental Tables [file mmc1.docx]

**Suppl. Table 1.** P-wave parameters analysis

| **Feature** | **Unit** | **Brugada patients**  **(mean ± std)**  **N = 89** | **Negative Ajmaline subjects**  **(mean ± std)**  **N = 44** | **p-value** |
| --- | --- | --- | --- | --- |
| **Amplitude_I** |  | 0.086 ± 0.039 | 0.081 ± 0.026 | 0.771 |
| **Amplitude_II** |  | 0.204 ± 0.061 | 0.184 ± 0.059 | 0.149 |
| **Amplitude_III** |  | 0.144 ± 0.059 | 0.122 ± 0.056 | **0.045** |
| **Amplitude_V1** |  | 0.088 ± 0.034 | 0.076 ± 0.024 | 0.058 |
| **Amplitude_V2** |  | 0.076 ± 0.028 | 0.078 ± 0.030 | 0.614 |
| **Amplitude_V3** | [mV] | 0.123 ± 0.034 | 0.115 ± 0.035 | 0.406 |
| **Amplitude_V4** |  | 0.116 ± 0.032 | 0.105 ± 0.032 | 0.166 |
| **Amplitude_V5** |  | 0.107 ± 0.032 | 0.093 ± 0.032 | 0.066 |
| **Amplitude_V6** |  | 0.098 ± 0.031 | 0.085 ± 0.032 | **0.044** |
| **Amplitude_aVF** |  | 0.170 ± 0.060 | 0.149 ± 0.058 | 0.104 |
| **Amplitude_aVL** |  | 0.067 ± 0.028 | 0.056 ± 0.018 | 0.066 |
| **Amplitude_aVR** |  | 0.140 ± 0.038 | 0.129 ± 0.034 | 0.166 |
| **Area_I** |  | 4.012 ± 2.045 | 3.691 ± 1.279 | 0.953 |
| **Area_II** |  | 10.055 ± 3.157 | 8.544 ± 3.243 | **0.024** |
| **Area_III** |  | 6.418 ± 3.214 | 5.123 ± 3.184 | **0.024** |
| **Area_V1** |  | 3.103 ± 1.454 | 2.296 ± 0.886 | **0.001** |
| **Area_V2** |  | 2.594 ± 1.459 | 2.453 ± 1.068 | 0.991 |
| **Area_V3** | au | 5.337 ± 1.747 | 4.721 ± 1.778 | 0.087 |
| **Area_V4** |  | 5.452 ± 1.715 | 4.617 ± 1.790 | **0.023** |
| **Area_V5** |  | 5.201 ± 1.690 | 4.279 ± 1.717 | **0.011** |
| **Area_V6** |  | 4.883 ± 1.628 | 3.934 ± 1.669 | **0.008** |
| **Area_aVF** |  | 8.101 ± 3.184 | 6.740 ± 3.231 | **0.028** |
| **Area_aVL** |  | 2.517 ± 1.456 | 1.967 ± 1.050 | **0.032** |
| **Area_aVR** |  | 7.015 ± 1.977 | 6.111 ± 1.795 | **0.043** |
| **Axis** | degree | 56.437 ± 47.501 | 61.317 ± 22.068 | 0.271 |
| **Complexity_I** |  | 5.158 ± 5.763 | 3.514 ± 3.723 | 0.085 |
| **Complexity_II** |  | 1.532 ± 1.146 | 1.730 ± 1.420 | 0.630 |
| **Complexity_III** |  | 3.202 ± 2.520 | 3.443 ± 2.349 | 0.551 |
| **Complexity_V1** | Number | 2.902 ± 2.105 | 3.045 ± 2.049 | 0.951 |
| **Complexity_V2** | Of | 4.273 ± 3.461 | 3.872 ± 2.314 | 0.557 |
| **Complexity_V3** | Peaks | 2.121 ± 1.813 | 2.071 ± 1.738 | 0.787 |
| **Complexity_V4** |  | 1.787 ± 1.381 | 2.103 ± 2.197 | 0.245 |
| **Complexity_V5** |  | 1.893 ± 1.864 | 1.994 ± 1.732 | 0.522 |
| **Complexity_V6** |  | 2.000 ± 1.935 | 2.068 ± 1.851 | 0.570 |
| **Complexity_aVF** |  | 1.936 ± 1.442 | 2.130 ± 1.624 | 0.695 |
| **Complexity_aVL** |  | 6.545 ± 4.773 | 5.949 ± 4.116 | 0.399 |
| **Complexity_aVR** |  | 1.634 ± 1.615 | 1.505 ± 1.103 | 0.341 |
| **Entropy_I** |  | 3.057 ± 0.124 | 3.088 ± 0.097 | 0.162 |
| **Entropy_II** |  | 3.124 ± 0.098 | 3.115 ± 0.087 | 0.682 |
| **Entropy_III** |  | 3.080 ± 0.113 | 3.073 ± 0.113 | 0.863 |
| **Entropy_V1** |  | 2.961 ± 0.163 | 2.948 ± 0.157 | 0.682 |
| **Entropy_V2** |  | 3.018 ± 0.132 | 2.997 ± 0.143 | 0.372 |
| **Entropy_V3** | a.u | 3.075 ± 0.119 | 3.055 ± 0.124 | 0.283 |
| **Entropy_V4** |  | 3.117 ± 0.106 | 3.101 ± 0.109 | 0.249 |
| **Entropy_V5** |  | 3.124 ± 0.089 | 3.114 ± 0.098 | 0.833 |
| **Entropy_V6** |  | 3.118 ± 0.088 | 3.118 ± 0.092 | 0.874 |
| **Entropy_aVF** |  | 3.100 ± 0.120 | 3.103 ± 0.092 | 0.968 |
| **Entropy_aVL** |  | 3.012 ± 0.145 | 2.997 ± 0.145 | 0.509 |
| **Entropy_aVR** |  | 3.116 ± 0.091 | 3.123 ± 0.083 | 0.926 |
| **FWHM** | [ms] | 50.959 ± 13.678 | 46.619 ± 10.842 | **0.043** |
| **PR Interval** | [ms] | 177.458 ± 24.827 | 170.335 ± 21.978 | 0.098 |
| **P-wave_Duration** | [ms] | 135.592 ± 17.369 | 124.207 ± 15.717 | **0.001** |
| **SampleEntropy_I** |  | 0.309 ± 0.178 | 0.288 ± 0.122 | 0.771 |
| **SampleEntropy_II** |  | 0.216 ± 0.057 | 0.244 ± 0.082 | 0.061 |
| **SampleEntropy_III** |  | 0.293 ± 0.128 | 0.343 ± 0.161 | 0.064 |
| **SampleEntropy_V1** |  | 0.246 ± 0.082 | 0.290 ± 0.100 | **0.016** |
| **SampleEntropy_V2** | a.u | 0.353 ± 0.157 | 0.342 ± 0.130 | 0.818 |
| **SampleEntropy_V3** |  | 0.237 ± 0.074 | 0.252 ± 0.100 | 0.536 |
| **SampleEntropy_V4** |  | 0.233 ± 0.064 | 0.265 ± 0.119 | 0.103 |
| **SampleEntropy_V5** |  | 0.229 ± 0.075 | 0.255 ± 0.094 | 0.106 |
| **SampleEntropy_V6** |  | 0.226 ± 0.077 | 0.256 ± 0.089 | **0.024** |
| **SampleEntropy_aVF** |  | 0.233 ± 0.073 | 0.272 ± 0.117 | 0.078 |
| **SampleEntropy_aVL** |  | 0.420 ± 0.201 | 0.430 ± 0.187 | 0.749 |
| **SampleEntropy_aVR** |  | 0.217 ± 0.066 | 0.237 ± 0.062 | 0.093 |
| **Terminal_Force_V1** | [mV ms] | 2.341 ± 1.470 | 1.631 ± 1.023 | **0.011** |

**Suppl. Table 2.** P-wave parameters comparison analysis between BrS patients with and without history of AF.

| **Feature** | **Unit** | **Brugada without AF**  **(mean ± std)**  N=79 | **Brugada with AF**  **(mean ± std)**  N=10 | **p-value** |
| --- | --- | --- | --- | --- |
| **Amplitude_I** |  | 0.086 ± 0.040 | 0.087 ± 0.032 | 0.365 |
| **Amplitude_II** |  | 0.203 ± 0.057 | 0.207 ± 0.089 | 0.272 |
| **Amplitude_III** |  | 0.144 ± 0.055 | 0.144 ± 0.095 | 0.301 |
| **Amplitude_V1** |  | 0.087 ± 0.035 | 0.093 ± 0.023 | 0.862 |
| **Amplitude_V2** |  | 0.074 ± 0.027 | 0.088 ± 0.028 | 0.995 |
| **Amplitude_V3** | [mV] | 0.123 ± 0.032 | 0.123 ± 0.048 | 0.393 |
| **Amplitude_V4** |  | 0.115 ± 0.030 | 0.121 ± 0.047 | 0.386 |
| **Amplitude_V5** |  | 0.106 ± 0.030 | 0.111 ± 0.047 | 0.533 |
| **Amplitude_V6** |  | 0.098 ± 0.029 | 0.101 ± 0.047 | 0.500 |
| **Amplitude_aVF** |  | 0.170 ± 0.056 | 0.170 ± 0.093 | 0.245 |
| **Amplitude_aVL** |  | 0.067 ± 0.027 | 0.072 ± 0.038 | 0.763 |
| **Amplitude_aVR** |  | 0.140 ± 0.037 | 0.143 ± 0.043 | 0.272 |
| **Area_I** |  | 3.992 ± 2.048 | 4.224 ± 2.014 | 0.492 |
| **Area_II** |  | 10.041 ± 2.872 | 10.201 ± 5.275 | 0.261 |
| **Area_III** |  | 6.429 ± 2.920 | 6.304 ± 5.399 | 0.251 |
| **Area_V1** |  | 3.129 ± 1.502 | 2.835 ± 0.756 | 0.211 |
| **Area_V2** |  | 2.527 ± 1.436 | 3.280 ± 1.520 | 0.734 |
| **Area_V3** | au | 5.312 ± 1.603 | 5.591 ± 2.830 | 0.686 |
| **Area_V4** |  | 5.434 ± 1.562 | 5.632 ± 2.852 | 0.372 |
| **Area_V5** |  | 5.185 ± 1.545 | 5.368 ± 2.772 | 0.295 |
| **Area_V6** |  | 4.876 ± 1.490 | 4.953 ± 2.672 | 0.267 |
| **Area_aVF** |  | 8.098 ± 2.900 | 8.134 ± 5.301 | 0.220 |
| **Area_aVL** |  | 2.488 ± 1.356 | 2.812 ± 2.225 | 0.658 |
| **Area_aVR** |  | 6.998 ± 1.868 | 7.199 ± 2.873 | 0.430 |
| **Axis** | degree | 56.002 ± 49.363 | 60.920 ± 19.458 | 0.639 |
| **Complexity_I** |  | 5.068 ± 5.746 | 6.093 ± 5.879 | 0.307 |
| **Complexity_II** |  | 1.545 ± 1.170 | 1.393 ± 0.854 | 0.125 |
| **Complexity_III** |  | 3.115 ± 2.450 | 4.100 ± 3.016 | 0.100 |
| **Complexity_V1** | Number | 2.923 ± 2.122 | 2.679 ± 1.913 | 0.923 |
| **Complexity_V2** | Of | 4.358 ± 3.534 | 3.393 ± 2.431 | 0.792 |
| **Complexity_V3** | Peaks | 2.144 ± 1.826 | 1.886 ± 1.658 | 0.780 |
| **Complexity_V4** |  | 1.824 ± 1.408 | 1.407 ± 0.988 | 0.583 |
| **Complexity_V5** |  | 1.908 ± 1.917 | 1.743 ± 1.184 | 0.207 |
| **Complexity_V6** |  | 2.002 ± 1.979 | 1.979 ± 1.422 | 0.445 |
| **Complexity_aVF** |  | 1.916 ± 1.448 | 2.136 ± 1.358 | 0.124 |
| **Complexity_aVL** |  | 6.471 ± 4.792 | 7.307 ± 4.520 | 0.653 |
| **Complexity_aVR** |  | 1.631 ± 1.617 | 1.664 ± 1.594 | 0.201 |
| **Entropy_I** |  | 3.053 ± 0.127 | 3.092 ± 0.090 | 0.379 |
| **Entropy_II** |  | 3.124 ± 0.092 | 3.122 ± 0.146 | 0.500 |
| **Entropy_III** |  | 3.082 ± 0.107 | 3.064 ± 0.163 | 0.492 |
| **Entropy_V1** |  | 2.953 ± 0.164 | 3.043 ± 0.128 | 0.0823 |
| **Entropy_V2** |  | 3.018 ± 0.130 | 3.022 ± 0.149 | 0.500 |
| **Entropy_V3** | a.u | 3.075 ± 0.118 | 3.077 ± 0.127 | 1.000 |
| **Entropy_V4** |  | 3.116 ± 0.104 | 3.122 ± 0.132 | 0.783 |
| **Entropy_V5** |  | 3.125 ± 0.083 | 3.117 ± 0.131 | 0.468 |
| **Entropy_V6** |  | 3.120 ± 0.087 | 3.101 ± 0.098 | 0.201 |
| **Entropy_aVF** |  | 3.102 ± 0.115 | 3.074 ± 0.160 | 0.320 |
| **Entropy_aVL** |  | 3.010 ± 0.147 | 3.037 ± 0.129 | 0.923 |
| **Entropy_aVR** |  | 3.115 ± 0.088 | 3.123 ± 0.121 | 0.667 |
| **FWHM** | [ms] | 50.986 ± 13.479 | 50.678 ± 15.633 | 0.964 |
| **PR Interval** | [ms] | 177.903 ± 25.102 | 172.871 ± 21.328 | 0.149 |
| **P-wave Duration** | [ms] | 136.452 ± 17.367 | 130.723 ± 14.746 | 0.250 |
| **SampleEntropy_I** |  | 0.304 ± 0.178 | 0.363 ± 0.171 | 0.091 |
| **SampleEntropy_II** |  | 0.214 ± 0.058 | 0.236 ± 0.042 | 0.094 |
| **SampleEntropy_III** |  | 0.285 ± 0.119 | 0.376 ± 0.181 | 0.080 |
| **SampleEntropy_V1** |  | 0.263 ± 0.082 | 0.277 ± 0.068 | 0.130 |
| **SampleEntropy_V2** | a.u | 0.359 ± 0.161 | 0.285 ± 0.087 | 0.852 |
| **SampleEntropy_V3** |  | 0.236 ± 0.074 | 0.243 ± 0.072 | 0.658 |
| **SampleEntropy_V4** |  | 0.231 ± 0.065 | 0.255 ± 0.046 | 0.131 |
| **SampleEntropy_V5** |  | 0.228 ± 0.077 | 0.246 ± 0.049 | 0.096 |
| **SampleEntropy_V6** |  | 0.225 ± 0.078 | 0.242 ± 0.066 | 0.110 |
| **SampleEntropy_aVF** |  | 0.230 ± 0.072 | 0.260 ± 0.075 | 0.102 |
| **SampleEntropy_aVL** |  | 0.418 ± 0.200 | 0.440 ± 0.208 | 0.667 |
| **SampleEntropy_aVR** |  | 0.214 ± 0.067 | 0.240 ± 0.050 | 0.069 |
| **Terminal_Force_V1** | [mV ms] | 2.406 ± 1.506 | 1.674 ± 0.763 | 0.067 |
